# Perception and Attitude of Medical Students towards Cadaveric Dissection in Anatomical Science Education

**DOI:** 10.4314/ejhs.v31i4.22

**Published:** 2021-07

**Authors:** Edmund Atta Asante, Raymond S Maalman, Mahamudu Ayamba Ali, Yaw Otchere Donkor, Joseph K Korpisah

**Affiliations:** Department of Basic Medical Sciences, School of Medicine, University of Health and Allied Sciences, Ho, Ghana; Department of Surgery, School of Medicine, University of Health and Allied Sciences, Ho, Ghana

**Keywords:** Cadaveric, Dissection, Medical students, Anatomical Science Education

## Abstract

**Background:**

Cadaveric dissection is essential and effective teaching method of anatomy in medical schools. In cadaveric dissection, the learner plays the central role of the teaching process and to view structures in their natural location. Cadaveric dissection is however perceived as tedious and time consuming by most students which influence their perception and attitude towards the teaching method. This study was therefore designed to evaluate UHAS medical students' perception and attitude toward dissection in the teaching and learning of human anatomy.

**Method:**

This was a cross-sectional and descriptive study. A Likert-style questionnaire, comprising 26 items was sent to study population via online using google form. Ethical issues were duly dealt with approval and consent.

**Result:**

Majority of the students (84.5%) perceived dissection more interesting, and the better way to really learn and understand the human body. About 87% of students also indicated that it assists in retention of what they learnt in theory. Majority of the students (74.5%) felt dissection should not be replaced by other forms of learning.

**Conclusion:**

There is a strong positive perception and attitude towards the use of cadaveric dissection as a teaching and learning method of anatomy. Cadaveric dissection brings about the skills, courageous and the ability to confidently work on the human body without any fear for future practice. It is therefore, recommended that more time should be allocated to cadaveric dissection.

## Introduction

Anatomy is one of the first basic and essential medical sciences in medical education (1). It lays the critical foundation for the study of other basic sciences and clinically oriented courses. An in-depth understanding of anatomy is therefore, imperative for crucial medical skills that include eliciting a clinical history and examination as well as clinical reasoning that would be required to making a correct diagnosis and the management of a patient (2). Thus, a sound knowledge of anatomy is essential for a safe and effective medical practice (3). Cadaveric dissection has been the bedrock of gross anatomy teaching and learning for years and forms a vital part of medical education (4,5). It significantly contributes to students understanding of anatomy (6,7). Anatomical education is experiencing the dilemma of embracing the reforms concerning innovations in medical education with technologies on one hand and finding avenues for effective utilization of the classical teaching methods like cadaveric dissection on the other. The framework of teaching anatomy pertaining to the curriculum and methods has been through a process of evolution in line with the demands of the medical profession (8). The worldwide trend in teaching at medical schools is characterized by a shift towards student-centered, integrated, clinical application models (9). As a result, there has been a significant reduction in the amount of time dedicated to traditional cadaveric dissection. This development has led to many researchers trying to find out how students of anatomy perceive these changes and their attitudes generally towards dissection despite the changes. Students experience the excitement during the dissection sessions, as most of them have never seen a dissected body or cadaver, and it makes them feel different from students of other professions (10).

However, some medical students do not consider dissection as the best method of teaching and learning anatomy (11) citing factors such as smell, nausea, and irritation, as well as psychological, such as stress, depression, and emotional trauma (12). Such students have suggested other ways of learning to replace dissection. Despite these challenges, anatomy educators still resort this method of teaching with a blend to modern teaching and learning techniques such as interactive lectures, USG, MRI, CT scans, laparoscopy, and virtual cadavers (13). However, these methods have their challenges such as cost and the skills needed for their utilization especially in developing countries including Ghana. Cadaveric dissection, therefore, remains the sole effective teaching method of anatomy without regards to the perception and attitude of students. This study is designed to investigate the perception and attitude of medical students toward cadaveric dissection in a typical Ghanaian-based medical school. It also assessed the preferences of medical students towards other innovative and complementary methods for teaching and learning of anatomy.

## Materials and Methods

**Study design and participants recruitment criteria**: The study was a cross-sectional study involving 161 students recruited among third to sixth-year medical students in the University of Health and Allied Sciences (UHAS). These classes were included because they had prior exposure or currently exposed to cadaveric dissection in their third year of study. First and second-year medical students and other students of UHAS were excluded as they had no exposure and experience with dissection at these levels. However, potential participants who desired not to participate were exempted.

**Sample size determination**: A sample size of 168 participants was determined based on Yamane's formula (1998) with 10% nonrespondent rate. Simple random sampling was used in the selection of participants.

**Data collection tool and procedure**: The data collection tool was a structured questionnaire made up of 26 Likert-style items regarding medical students' perception and attitude towards cadaveric dissection. The items were closed-ended and addressed five broad areas with eight items evaluating positive experiences, seven items evaluating negative experiences, four on emotional effect whereas three and four items evaluating attitudes and acceptability of dissection, respectively.

The questionnaire was converted into an online google form and the link sent to targeted study population emails and other social medial platforms for responses. Reminders were sent periodically to participants and after two weeks a total of 161 responses were received.

**Statistical analysis**: Data were downloaded and organized in Microsoft Excel® spreadsheets and analysed using Statistical Package for the Social Sciences (SPSS) Inc., Chicago, version 22.0 for Windows. Data were analysed using descriptive and inferential statistic methods. P value <0.05 was considered to have statistical significance.

**Ethical issues**: Ethical clearance for the study was obtained from the UHAS Research Ethical Committee on 09/02/2020 with a reference number A9 (49)-19-20. Participants were informed that participation was voluntary. The form also had a section that respondents must indicate their willingness to participate in the study. No specific identifiers such as name and identification numbers were collected.

## Results

**Socio-demographic characteristics of respondents**: Table 1 shows that out of the 161 respondents, majority 116 (72.0%) were males. The level 600 was the least with 19% and level 300 students were 31.1%, and most 85 (52.8%) of respondents were between the age range of 24–29 years. Out of the total, 153 (95%) were Christians.

**Table 1:** Demographic characteristics of study participants (N=161)

Variables	Frequency	Percent
Sex		
Male	116	72.0
Female	45	28.0
Level		
300	50	31.1
400	43	26.7
500	37	23.0
600	31	19.0
Age in years		
18–23	73	45.3
24–29	85	52.8
30–35	2	1.2
36–40	1	0.6
Religion		
Christian	153	95.0
Muslim	5	3.1
Others	3	1.9

N: Sample size; (%): percentages

**Positive perception of students on cadaveric dissection teaching-learning method of anatomy**: Table 2 shows the variables framed to determine whether respondents agree or disagree with statements perceived as positive concerning dissection by selecting strongly agree or agree as against strongly disagree or disagree respectively. Majority of the respondents agreed with all variables evaluating the positive aspect of dissection. Dissection helped students to recall what had been learnt (87%), makes learning interesting (84.5%), deepens my understanding (90.1%), provided a 3-dimensional perspective (83.2%).

**Table 2:** Positive perception of students toward cadaveric dissection as learning tool

Variable	SA	A	N	D	SD
	f(%)	f(%)	f(%)	f(%)	f(%)
My first visit was exciting	72(44.7)	38(23.6)	35(21.7)	9(5.6)	7(4.3)
Dissection deepened my understanding	88(54.7)	57(35.4)	9(5.6)	5(3.1)	2(1.2)
The dissection enhanced my respect towards the human body	72(44.7)	44(27.3)	26(16.1)	14(27.3)	5(3.1)
Provided better understanding of the effect of trauma	54(33.5)	59(36.6)	29(18.0)	18(11.2)	1(0.6)
Dissection makes learning more interesting	74(46.0)	62(38.5)	10(6.2)	11(6.8)	4(2.5)
The dissection helped me to recall what I learnt	69(42.9	71(44.1)	10(6.2)	6(3.7)	5(3.1)
Gives me a lasting knowledge	62(38.5)	59(36.6)	26(16.1)	9(5.6)	5(3.1)
Provides a three-dimensional perspective of the structures	58(36.0)	76(47.2)	11(6.8)	8(5.0)	8(5.0)

f: frequency; (%): percentages; SA: strongly Agree; A: Agree; N: Neutral; D: disagree; SD: Strongly disagree

**Negative perception of students on cadaveric dissection as a teaching-learning method of anatomy**: Table 3 represents the evaluation of whether respondents agree or disagree with statements about dissection considered as negative by selecting strongly agreed or agreed as against strongly disagreed or disagreed respectively. As high as 92.5% either strongly agreed or agreed to the statement, ‘I did not like the smell of formalin’. Besides, 84.5% either strongly agreed or agreed to the statement, ‘dissection is time-consuming’. However, 90.7% either strongly disagreed or disagreed that dissection is against their religion or culture (88.8%). Dissection was very stressful for 80.2% of medical students as 56.5% found it difficult locating the structures.

**Table 3:** Negative perception of students on cadaveric dissection as a teaching and learning tool

variables	SA f(%)	A f(%)	N f(%)	D f(%)	SA f(%)
It was difficult locating structures	25(15.5)	66(41.0)	25(15.5)	40(24.8)	4(2.5)
Dissection was stressful	79(49.1)	50(31.1)	17(10.6)	10(6.2)	5(3.1)
I could not differentiate between structures	13(8.1)	43(26.7)	31(19.3)	68(42.2)	6(3.7)
It was time consuming	85(52.8)	51(31.7)	10(6.2)	8(5.0)	7(4.3)
I did not like the smell of formalin	115(71.4)	34(21.1)	6(3.7)	1(0.6)	5(3.1)
I feel dissection is against my culture	2(1.2)	1(0.6)	15(9.3)	44(27.3)	99(61.5)
I feel dissection is against my religion	2(1.2)	2(1.2)	11(6.8)	39(24.2)	107(66.5)

f: frequency; (%): percentages; SA: strongly Agree; A: Agree; N: Neutral; D: disagree; SD: Strongly disagree

**Emotional impact of cadaveric dissection on medical students**: Table 4 shows that out of the 161 respondents, 60 (37.3%) participants either strongly agreed or agreed to have anxiety before, during and after dissection. One-third (30.1%) of medical students were not mentally prepared for dissection. About one-half (51%) of medical students have had a prior experience with the dead body which helps 72 (44.7%) to adjust to such exposure to cadaveric dissection.

**Table 4:** Emotional impact of cadaveric dissection on medical students

Variables	SA f(%)	A f(%)	N f(%)	D f(%)	SD f(%)
I had anxiety before during and after my first dissection	29(18.0)	31(19.3)	14(8.7)	60(37.3)	27(16.8)
I prepared mentally for dissection	28(17.4)	47(29.2)	37(23.0)	30(18.6)	19(11.8)
I had a prior exposure to a dead body	36(22.4)	46(28.6)	8(5.0)	37(23.0)	34(21.1)
The prior exposure helped me	40(24.8)	32(19.9)	22(13.7)	38(23.4)	29(18.0)

f: frequency; (%): percentages; SA: strongly Agree; A: Agree; N: Neutral; D: disagree; SD: Strongly disagree

**Acceptability and attitude towards cadaveric dissection**: Table 5 shows that the majority of respondents viewed dissection as an effective teaching and learning method for anatomy. As high as 77.6% either strongly agreed or agreed that they will be a disadvantage for not attending dissection sessions. Also, Majority of the students were regular attendants of dissection (86.9%), recognized cadaver as once human (96.3%).

**Table 5:** Acceptability of cadaveric dissection as a method of teaching and learning anatomy and students' general attitude towards dissection

Variables	SA(f%)	A f(%)	N f(%)	D f(%)	SD f(%)
I prefer dissection over other forms of learning anatomy	10(6.2)	16(9.9)	39(24.2)	68(42.2)	28(17.4)
I will be disadvantaged if I do not attend dissection	76(47.2)	49(30.4)	21(13.0)	10(6.2)	5(3.1)
More time should be allocated to dissection	63(39.1)	35(21.7)	34(21.1)	20(12.4)	9(5.8)
Dissection should be replaced by lectures, prosections	14(8.7)	10(6.2)	17(10.6)	49(30.4)	71(44.1)
I know cadaver was once a human like me	112(69.6)	43(26.7)	2(1.2)	0	4(2.5)
I attend dissection regularly	115(71.4)	25(15.5)	10(6.2)	6(3.7)	5(3.1)
I have respect and empathy for the cadaver	79(49.1)	57(35.4)	17(10.6)	5(3.1)	3(1.90

f: frequency; (%): percentages; SA: strongly Agree; A: Agree; N: Neutral; D: disagree; SD: Strongly disagree

**Factors that influence having anxiety during dissection**: Table 6 shows the relationship between sex, religion before exposure to a dead body and anxiety. The results demonstrate no association between sex, religion, and anxiety (p value=0.395, 0.702 respectively). However, there is an association of statistical significance between prior exposure to a dead body and anxiety (0.000).

**Table 6:** Relationship between sex, religion, prior exposure to a dead body and anxiety

Variables	Anxious f(%)	Not anxious f(%)	Neutral f(%)	P value
Sex				
Male	43(31)	65(56)	8(7)	0.395
Female	17(38)	22(49)	6(13)	
Religion				
Christian	58(38)	81(53)	14(9)	0.702
Muslim	2(40)	3(60)	0	
Others	0	3(100)	0	
Prior exposure				
Yes	9(11)	65(79)	8((9.8)	0.00
No	57(80.3)	7(9.9)	7(9.9)	
Neutral	1(12.5)	0	7(87.5)	

f: frequency; (%): percentages

## Discussion

**Students' perception towards cadaveric dissection**: The students had a strong belief that dissection makes learning more interesting (84.5%), it makes them recall what they learn (87%) and provides the 3-dimensional perspective of structures (83.2%). Cadaveric dissection also deepens their understanding (90.1%), gives them a lasting knowledge (75.1%) and enhances their respect for the human body (72%). There was also a general description of the first dissection experience as very exciting (68.3%). This positive perception expressed by the students is similar to those expressed in a study by Sharma and Gupta (14). Dissabandara *et al.*, (2) had observed in a study conducted in Australia that majority of students (>75%) agreed with all the survey instrument items that reflected positive perceptions of cadaveric dissections. The present study also corroborated a study conducted by Izunya *et al.*, (15) were about 90% of the students recognized dissection as indispensable as far as the study of anatomy is concerned. Despite this digital age, with the advanced technology of teaching anatomy using 3-dimensional imaging equipment such as anatomagetable, these are very expensive for resource-deprived countries such as Ghana. The cadaver also served as the first patient to the medical doctor under training making them familiarise with the dead body before their clinical years.

Nevertheless, students in this current study expressed major dissatisfaction with some aspects of dissections. They did not like the smell of formalin (92.5%), had challenges with identifying structures (56.5%) and considered dissection as stressful (80.2%) and time-consuming (84.5%). This finding is consistent with other studies (10,16,17). In a study by Dissabandara *et al.*, (2) a significant number of respondents also, related to items that reflected a negative perception of cadaveric dissection including “being time-consuming,” (59.3%) “difficult to identify structures” (48.4%), and “do not like the smell of preservatives” (45.1%). Another study conducted at the University of Development Studies, affirms the negative experiences of dissection observed in this present study (18). In the preservation of cadavers for dissection, the most used chemical agent is the formaldehyde as it hardens proteins and prevents them from decomposition (19). However, formaldehyde has a sharp odour that can be detectable at low concentrations, and its vapour and solutions are known as skin and eye irritants in human beings. The common effects of formaldehyde exposure are various symptoms caused by irritation of the mucosa in the eyes and upper respiratory tract and these are experienced by the students during cadaveric dissection. Formaldehyde is as well emphasized as carcinogenic by experimental studies (19, 20, 21) and has harmful effects on many systems such as the respiratory system, nervous system, and digestive system. There is therefore a dire need for anatomists and scientists to determine better means of preservation of cadavers that minimizes these harmful effects.

Dissection in its current form is time-consuming and stressful compared to other forms of learning anatomy; but this slow but systematic nature of dissections is beneficial to study and understand sophisticated anatomical structures such as the brain, neck, and limbs which can be very challenging for a novice.

**Students' attitude towards cadaveric dissection**: The majority were regular attendants of dissection (86.9%), had respect and empathy for the cadaver (84.5%) and recognized the cadaver as once human (96.3%). The results of this current study reinforce cadaveric dissection as a very important tool in the teaching and learning of anatomy. This result is also consistent with studies conducted by Dissabandara *et al.*, (2) and Abass and Saeed, (22), where the majority of the respondents were regular attendants of dissection, had respect and empathy for the cadaver and recognized the cadaver as once human. Medical students are usually enthusiastic in using cadavers to learn the structures of the human body. They are aware that these are real human bodies that were once living like them and the patient they will be working on in their future career as medical doctors. This feeling teaches medical students the gift of empathy on their patients better than the use of machines for learning anatomy.

For instance, Weeks *et al.*'s (23) attest that cadaveric dissection offers an opportunity to medical students to develop a relationship between them and the cadaver donor which has been coined to be a model of doctor-patient relationship at an early stage of the medical training. Medical students should be exposed to opportunities that inculcate professional attributes essential for medical practice such as respect, dignity and compassion. Dissection, therefore, provides students with such opportunities to learn to be respectful and develop compassion and empathy towards suffering.

**The emotional impact of dissection on medical students**: With regards to emotional effects of dissection on the students, more than one-half of the students had no anxiety before, during and after dissection with 46.6% preparing mentally for dissection. The present study findings did not agree with previous studies where students did experience anxiety which impacted negatively on their learning of anatomy (12, 24,25,26). For medical students starting the first year of medical school, cadaveric dissection can be a rite of passage that lives up to its name. Often, it is the first-time students come into contact with a dead human body, and it can be a harrowing experience, and many times students react to the discomfort by being emotionally anxious.

The anxiety of students can be influenced by factors such as sex, religion, cultural believes and practices regarding the dead body. In this study, there was, however, no relationship between sex and anxiety though previous studies found males to be less likely to experience anxiety before, during and after dissection (24,27,28,29). This study also shows no association between religion and anxiety. This affirms a study conducted by Shalev and Nathan (30). In that study, it was found that Jewish and non-Jewish medical students did not differ significantly in the amount of anxiety experienced due to dissection. This observation, however, contradicts an earlier study by Aday (31) where it was observed that Christians who attended church more often had lower death anxiety scores. It was then postulated that death and the anxiety associated with dying is lower in people who participate more often in religious activities since their lives are more focused on a spiritual level. In our current study, however, having prior exposure to a dead body had a significant (p=0.000) association with the development of a coping mechanism and hence less anxiety during dissection.

**Acceptability of cadaveric dissection by students**: Generally, the students considered dissection as inevitable. Most of the respondents claimed they would be disadvantaged if they did not attend dissection (77.6%) with 60.8% asking for more time for dissection. Also, a significant number (74.5%) disagreed to the replacement of dissection with other methods of learning and teaching anatomy. This finding validates other studies which recognized dissection as very acceptable means of teaching and learning anatomy with only a few students calling for its replacement (2,15,18,31).

Only a few numbers of students (16.1%) agreed that dissection classes were preferred over other forms of learning anatomy. This is also in support of a study conducted by Dissabandara *et al.*, (2) where only about 36% preferred dissection over other forms of learning. This illustrates that though dissection is very essential, other innovative ways of learning must be allowed to supplement the teaching and learning of anatomy (32,33).

This study has demonstrated that medical students have a strong positive perception and attitude towards the use of cadavers in the teaching and learning of anatomy. It supports a definitive role cadaveric dissection play in the teaching and learning of anatomy in medical education. Medical students have a high preference for cadaveric dissection as it offers them much understanding of the subject. Challenges such as smell from the preservatives (formaldehyde), being stressful, and time-consuming were outlined from the study. There is no emotional difference of students towards cadavers as sex is concerned. Cadaveric dissection brings about the skills, courageous and the ability to confidently work on the human body without any fear for future practice.

The study was limited to only one university which has been newly established with huge resource challenges and that might influence the perception and attitude of the students towards the dissections. The study should have included students from other universities to prevent biases. The strength of the study is that it made use of students who had experienced with the dissection, either at the time of the study or before the study.

We recommend that human dissection should be an integral part of the medical training, therefore, the department of anatomy should allocate adequate time for dissection. It is also recommended that a course on death and dying should be introduced in the first year to expose students early enough to ensure the development of a coping mechanism.

Lastly, other alternative methods such as plastic models, prosected specimens, animation, and painting, are recommended to complement dissection.
